# Predicting the Severity of Acute Appendicitis of Young Children (<3 Years Old): Development and Assessment of a New Prediction Nomogram

**DOI:** 10.3389/fped.2021.763125

**Published:** 2021-11-18

**Authors:** Yang Chen, Zhiyong Wang, Dong Xiao, Hongwu Zeng, Xiaopeng Ma

**Affiliations:** ^1^Shenzhen Children's Hospital, Shenzhen, China; ^2^College of Medicine, Shantou University, Shantou, China

**Keywords:** complicated appendicitis, young children, laboratory examination, retrospective analysis, prediction model

## Abstract

**Objective:** There is a lack of assessment methods of acute appendicitis in little children. The purpose of this study was to develop and internally validate a nomogram for predicting the severity of acute appendicitis of young children (<3 years old).

**Methods:** We develop a prediction model based on a training dataset of 121 patients (<3 years old) with acute appendicitis. Admission information was collected between January 2010 and January 2021, which contained demographic characteristic, laboratory examinations, treatment and pathology type, etc. Logistic regression analysis was used to identify independent risk factors and establish the predictive model. C-index and calibration curves were applied to evaluate the performance of the nomogram. Then corrected C-index was calculated to conduct internal verification by using the bootstrapping validation. Decision curve analysis determined clinical application of the prediction model.

**Results:** Predictors contained in the prediction nomogram included weight for age, onset time (from developing symptoms to hospital), admission temperature, leukocyte count, neutrophil ratio, and total bilirubin. Logistic regression analysis showed that weight for age (X1) < -2.32 SD (*P* = 0.046), onset time (X2) > 2.5 days (*P* = 0.044), admission temperature (X3) > 38.5°C (*P* = 0.009), leukocyte count (X4) > 12.185^*^109/L (*P* = 0.045), neutrophil ratio (X5) > 68.7% (*P* = 0.029), and total bilirubin (X6) > 9.05 μmol/L (*P* = 0.035) were found to be significant for predicting the severity of appendicitis. The logistic regression equation was logit (*P*) = −0.149X1 + 0.51X2 + 1.734X3 + 0.238X4 + 0.061X5 + 0.098X6 – 75.229. C-index of nomogram was calculated at 0.8948 (95% Cl: 0.8332–0.9567) and it still was 0.8867 through bootstrapping validation. Decision curve analysis showed that when the threshold probability ranged from 14 to 88%, there is a net benefit of using this prediction model for severity of appendicitis in little children.

**Conclusion:** This novel nomogram incorporating the weight for age, onset time, admission temperature, leukocyte count, neutrophil ratio, and total bilirubin could be conveniently used to estimate the severity of appendicitis of young children <3 years old) and determine appropriate treatment options in time.

## Introduction

Acute appendicitis (AA) is one of the common abdominal surgical emergencies among children. The symptoms and signs of acute appendicitis in young children (<3 years old) are often unobvious because of their unstable emotions, communication problems, and uncooperativeness during examination. The imaging characteristics are limited by insensitive inflammatory reactions and movable appendix. The early assessment of acute appendicitis in this age group remains a huge challenge because of nonspecific presentations. However, the damage caused by this disease is enormous and even life-threatening, such as perforation and sepsis (1–3).

Treatments of AA include conservative medication and emergent appendectomy on admission. Because of the high rate of misdiagnosis, in-patient close clinical observation and repeat evaluations are commonly applied, during which children may suffer unnecessary pain, and cost increase for families, especially for patients who had an operation after conservative treatment failed (4). But for pediatric surgeons, the current evaluation systems, such as the appendicitis inflammatory response (AIR) score and the Alvarado score, are not satisfactory for young children (<3 years old) because of atypical symptoms and uncooperativeness during physical examinations. The surgical decision is based on the clinical experience of the pediatrician (5, 6).

It is generally believed that complicated appendicitis shows more severe appendicitis and tends to require surgery (7–10). Therefore, we performed a retrospective analysis to identify the risk factors of the clinical characteristics and develop a predictive model to assess the severity of AA in young children (<3 years old).

## Statistics and Methods

### Statistics

Research approval was obtained from the Ethics Committee of Shenzhen Children's Hospital (approval no. 2021059). We collected the information of young children (<3 years old) with appendicitis from January 2010 to 2021 in Shenzhen Children's Hospital. Those cases with other underlying diseases, secondary appendicitis, or were treated before hospitalization were excluded. According to the highly reliable contents in the records, we designed a catalog that included the gender, age, weight, onset time (from the development of symptoms to hospitalization), admission temperature, various laboratory indicators at admission, treatment, and postoperative pathological types. Considering that the weight of children correlates with premature delivery and increases with age, we calculated the standard deviation (SD) of the weight for age in order to compare the weight at different ages on the basis of the WHO database.

### Classification

According to the therapeutic effect and pathological manifestations, acute appendicitis can be divided into uncomplicated and complicated appendicitis. Uncomplicated appendicitis is when conservative treatment is successful or the pathological type is pure appendicitis. Complicated appendicitis is characterized by failure of conservative treatment, purulent appendicitis, gangrene perforated appendicitis, and periappendiceal abscess (7, 11).

### Statistical Methods

Statistical analysis was performed using the R software (version 3.1.1). Univariate logistic regression was performed to identify the potential risk factors for young patients (<3 years old) with AA. Then, multivariable logistic regression analysis was used to build a predictive model by incorporating the features selected previously. The risk factors were considered as odds ratio (OR) with 95% confidence interval (CI) and *p*-value. Statistical significance levels were all two-sided. All potential predictors were applied to develop a predictive model for the severity of acute appendicitis using the study cohort. Calibration curves were plotted to assess the calibration of the nomogram. To quantify the discrimination performance of the nomogram, Harrell's concordance index (C-index) was measured. The nomogram was subjected to bootstrapping validation (1,000 bootstrap resamples) to calculate a relative corrected C-index. A decision curve analysis was conducted to determine the clinical usefulness of the nomogram by quantifying the net benefits at different threshold probabilities in AA patients (<3 years old). The net benefit was calculated by subtracting the proportion of all patients who were false positives from the proportion of patients who were true positives and by weighing the relative harm of forgoing interventions compared with the negative consequences of an unnecessary intervention.

## Results

### General Information

A total of 121 cases (<3 years old) of appendicitis were admitted into Shenzhen Children's Hospital, accounting for 4.23% of all children (<14 years old) with appendicitis, which included 14 cases of children aged 0–1 years, 24 cases aged 1–2 years, and 83 cases aged 2–3 years. There were 28 cases of uncomplicated appendicitis (20 males and 8 females; mean age = 25.9 ± 8.4 months, range = 8–34 months) and 93 cases of complicated appendicitis (56 males and 37 females; mean age = 22.4 ± 10.6 months, range = 6–33 months). All data of patients, including demographic and clinical data, in the two groups are given in Table 1.

**Table 1:** Comparison of admission information between the two groups.

**Item**	**Uncomplicated**	**Complicated**	**χ^**2**^/*t***	***p*-value**
Gender (M/F)	20/8	56/37	1.158	0.282
Age (months)	22.4 ± 10.6	25.9 ± 8.4	−1.811	0.073
Weight for age, SD	0.13 ± 3.34	−2.92 ± 4.16	5.521	0.010
Onset time (days)	2.25 ± 1.43	3.04 ± 1.37	−2.595	0.013
Temperature (°C)	37.86 ± 0.56	38.35 ± 0.55	−4.071	<0.001
Leukocyte count ( × 10^9^/L)	11.79 ± 3.29	14.36 ± 3.63	−3.552	0.001
Neutrophil count ( × 10^9^/L)	10.05 ± 10.15	12.27 ± 8.84	−1.042	0.304
Neutrophil ratio (%)	68.41 ± 9.19	74.67 ± 12.55	−2.886	0.005
Platelet ( × 10^9^/L)	429.7 ± 186.1	355.7 ± 165.3	2.015	0.046
CRP (mg/L)	55.5 ± 34.33	75.98 ± 44.52	−2.239	0.027
PCT (ng/L)	4.62 ± 3.86	6.33 ± 3.79	−2.079	0.040
Indirect bilirubin (μmol/L)	1.98 ± 0.65	2.7 ± 2.68	−1.419	0.016
Direct bilirubin (μmol/L)	9.03 ± 6.4	11.7 ± 8.39	−1.549	0.012
Total bilirubin (μmol/L)	11.01 ± 6.65	14.4 ± 10.5	−1.614	0.011

*CRP, C-reactive protein; PCT, procalcitonin*.

### Univariate Logistic Regression Analysis

The above variables were filtrated by univariate logistic regression. Independent factors closely related to the severity of appendicitis included age, weight for age, onset time, admission temperature, leukocyte count, neutrophil ratio, C-reactive protein (CRP), procalcitonin (PCT), and total bilirubin. Sex, age, neutrophil count, and direct and indirect bilirubin were excluded (*p* > 0.5). The ORs and 95%CI are shown in Table 2.

**Table 2:** Univariate logistic regression analysis of various risk factors.

**Variables**	**OR**	**95%CI**	***p*-value**
Gender	0.545	0.116–2.558	0.282
Age (months)	0.847	0.686–1.044	0.072
Weight for age	1.181	0.977–1.427	0.001
Onset time (days)	2.127	1.220–3.707	0.009
Temperature (°C)	4.118	1.798–6.928	<0.001
Leukocyte count ( × 10^9^/L)	1.235	0.896–1.701	0.001
Neutrophil count ( × 10^9^/L)	1.095	0.979–1.225	0.260
Neutrophil ratio (%)	1.087	0.996–1.186	0.016
CRP (mg/L)	1.009	0.989–1.029	0.027
PCT (ng/L)	0.959	0.792–1.161	0.039
Indirect bilirubin (μmol/L)	0.899	0.677–1.013	0.122
Direct bilirubin (μmol/L)	0.625	0.425–0.732	0.156
Total bilirubin (μmol/L)	0.701	0.584–0.818	0.024

*OR, odds ratio; CRP, C-reactive protein; PCT, procalcitonin*.

### Multivariate Regression Analysis and the Development of a Predictive Model

**Figure 1:**
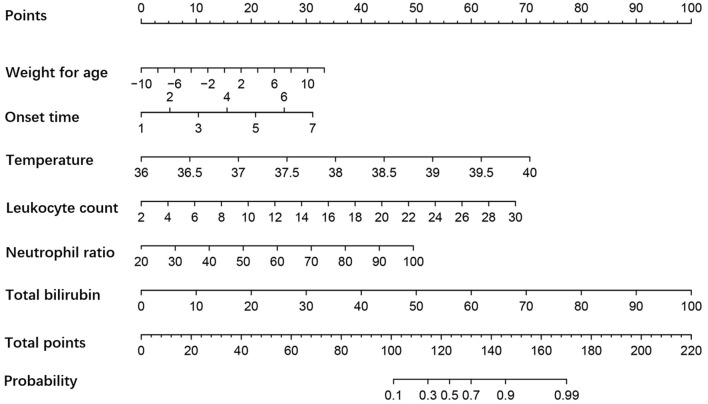
Nomogram of the severity of appendicitis in young children. The influencing effects of factors are shown in appropriate scale. The sum of all scores indicates the probability of complicated appendicitis, and more points suggest a more severe appendicitis.

According to the results in Table 2, multivariate regression analysis was applied to identify the six variables that were incorporated in the predictive model: weight for age, onset time, admission temperature, leukocyte count, neutrophil ratio, and total bilirubin. The following logistic regression equation was obtained: logit(*P*) = −0.149*X*_1_ + 0.51*X*_2_ + 1.734*X*_3_ + 0.238*X*_4_ + 0.061*X*_5_ + 0.098*X*_6_ – 75.229, where *X*_1_ is the weight for age (>-2.32 SD = 0 or < -2.32 SD = 1), *X*_2_ is the onset time (<2.5 days = 0 or >2.5 days = 1), *X*_3_ the admission temperature (<38.5°C = 0 or >38.5°C = 1), *X*_4_ the leukocyte count (<12.185 × 10^9^/L = 0 or >12.185 × 10^9^/L = 1), *X*_5_ is the neutrophil ratio (<68.7% = 0 or >68.7% = 1), and *X*_6_ is the total bilirubin (<9.05 μmol/L = 0 or >9.05 μmol/L = 1; see Table 3). The model was presented as a nomogram (Figure 1) according to the above independent predictors.

**Table 3:** Multivariate regression analysis of significant risk factors.

**Item**	***B***	**Standard errors**	**Wald**	***p*-value**	**Exp(*B*) (95%Cl)**
Weight (*X*_1_)	−0.149	0.017	3.965	0.046	0.968 (0.937–0.999)
Onset time (*X*_2_)	0.51	0.253	4.055	0.044	1.666 (1.014–2.737)
Temperature (*X*_3_)	1.734	0.66	6.904	0.009	5.661 (1.553–20.63)
Leukocyte count (*X*_4_)	0.238	0.119	4.003	0.045	1.269 (1.005–1.603)
Neutrophil ratio (*X*_5_)	0.061	0.028	4.759	0.029	1.063 (1.006–1.122)
Total bilirubin (*X*_6_)	0.098	0.047	4.449	0.035	1.103 (1.007–1.208)
Constant	−75.229	25.672	8.587	0.003	–

### Discrimination and Calibration

**Figure 2:**
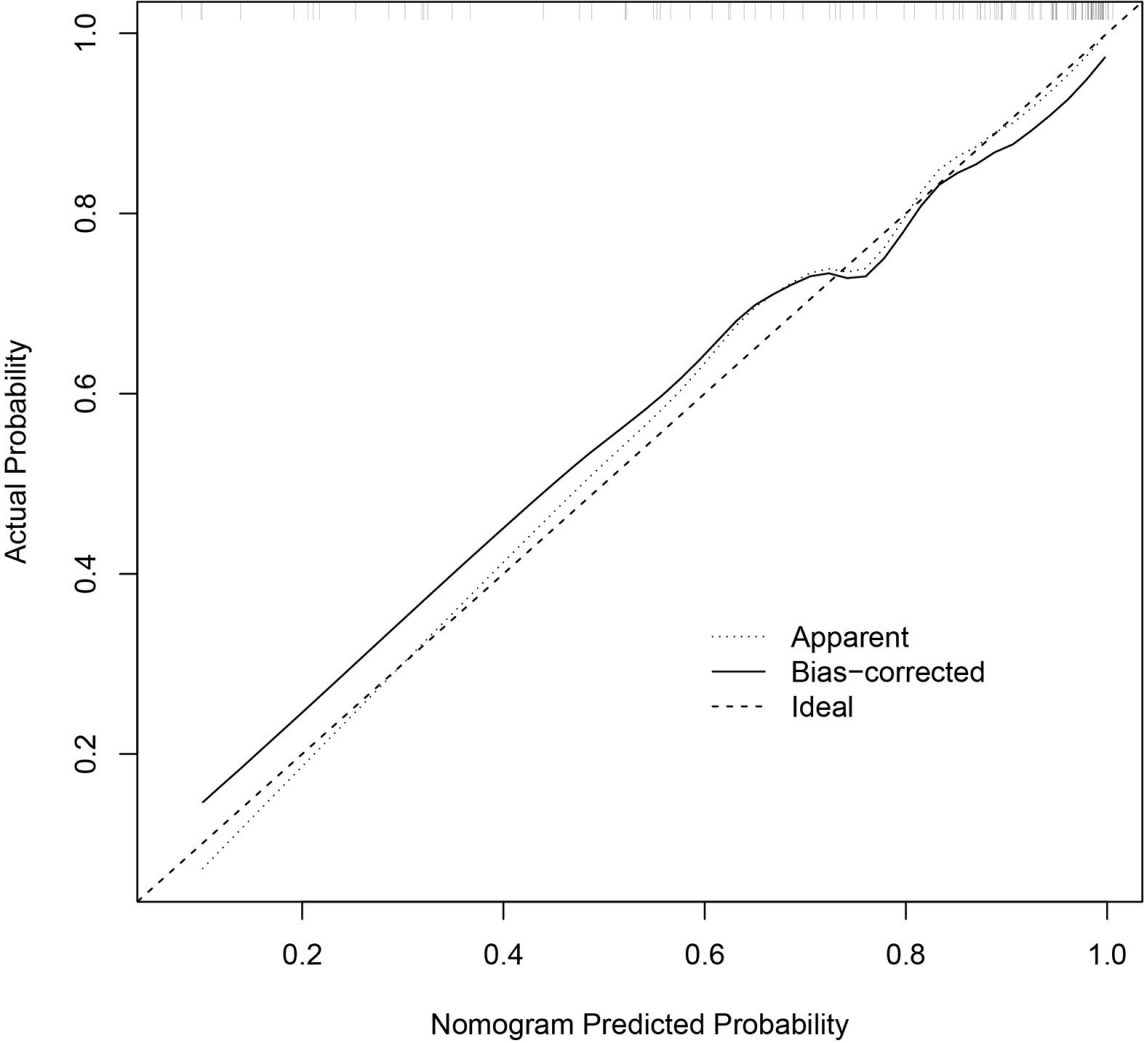
Calibration curves of the nomogram prediction. The *x*-axis and the *y*-axis represent the nomogram prediction and the actual situation, respectively. The *diagonal line* shows that the forecast is exactly what happened. The more the *solid line* matches the *diagonal*, the better the predictive accuracy.

The C-index for the predictive nomogram was 0.8948 (95% CI = 0.8332–0.9567). To verify the accuracy of the model, a corrected C-index was calculated through 1,000 bootstrap resamples, with a value of 0.8867. Meanwhile, the calibration curves indicated that the forecast was in good agreement with the actual situation (Figure 2). The results showed that the model addressed great predictive capability.

### Clinical Application

**Figure 3:**
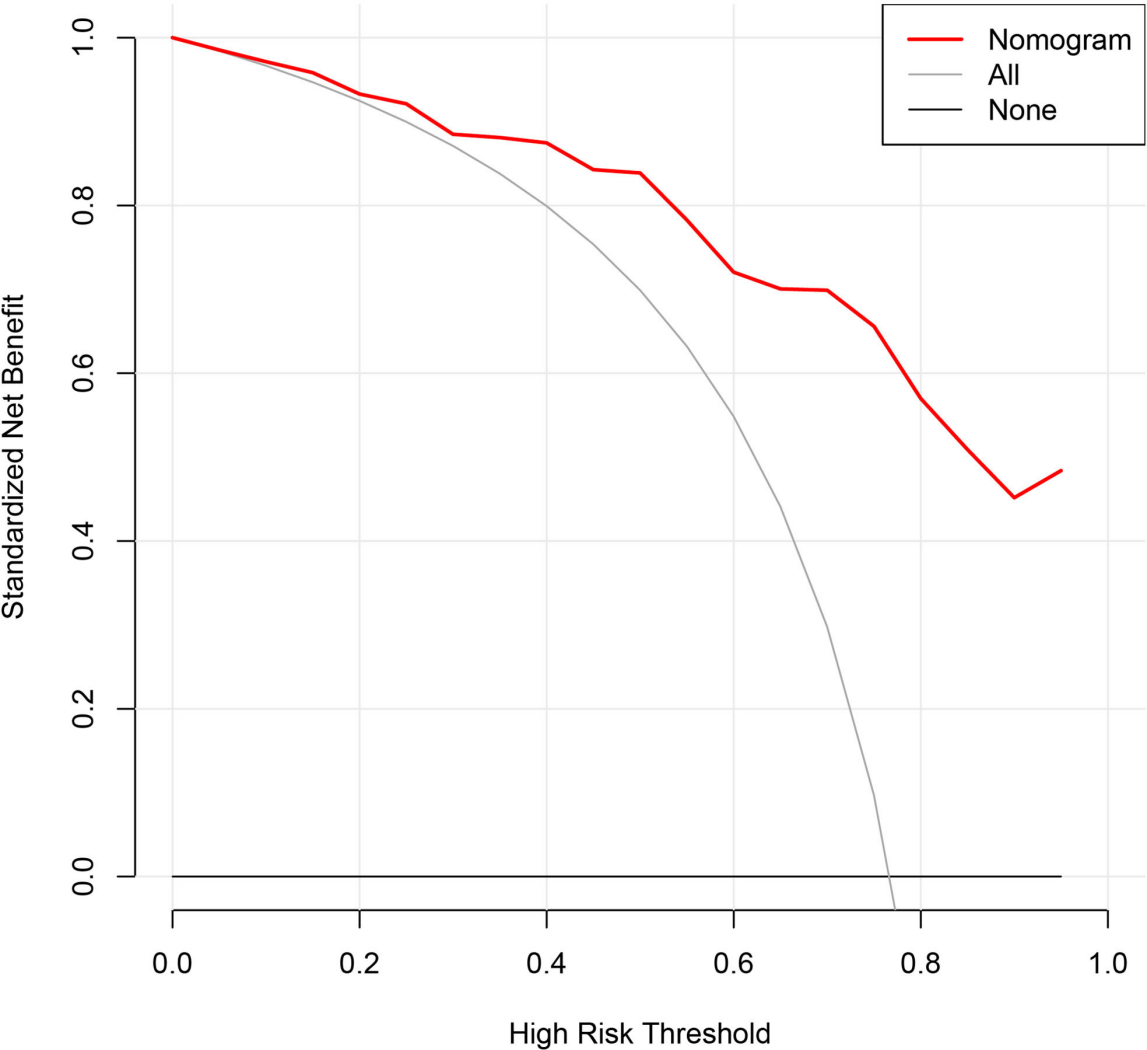
Decision curve analysis for the nomogram. The *x*-axis represents the threshold probability and the *y*-axis measures the net benefit. When the threshold probability ranges from 14 to 88%, there is a net benefit to using this predictive model for the severity of appendicitis in young children.

Furthermore, the decision curve analysis for the nomogram showed that there is a net benefit to using this predictive model for the severity of appendicitis in young children (<3 years old) when the threshold probability is between 14 and 88% (Figure 3).

## Discussion

Currently, the commonly used appendicitis scoring systems are the AIR score, the Alvarado score, or the pediatric appendicitis score (PAS), which are applied to evaluate possible appendicitis. A meta-analysis of the three scoring systems showed that the AIR score has the best diagnostic accuracy in children. Regrettably, the misdiagnosis rate is unacceptable for young children because pediatricians cannot elicit an effective response (5, 6). Studies on the inflammatory response suggested that further laboratory examination may facilitate the assessment of severity (7). Clinicians, especially in primary hospitals, have demanded for a new evaluation system in order to make a timely decision.

In our study, we established a predictive model for young children (<3 years old) using accurate admission information. The results indicated that the infection index of complicated appendicitis characterized by gangrene, perforation, and periappendiceal abscess was obviously higher than that of uncomplicated appendicitis. Weight for age < -2.32 SD (*p* = 0.046), onset time >2.5 days (*p* = 0.044), admission temperature >38.5°C (*p* = 0.009), leukocyte count >12.185 × 10^9^/L (*p* = 0.045), neutrophil ratio >68.7% (*p* = 0.029), and total bilirubin >9.05 μmol/L (*p* = 0.035) were closely related to the severity of appendicitis. A higher predictive probability means a more severe appendicitis and that the pediatrician should consider surgery more (8–10). To our knowledge, this is the first evaluation system especially for young children.

Not surprisingly, body temperature and leukocyte count were included into the model, which are usually included into common scoring systems and have been shown in many studies to be strongly associated with the severity of appendicitis (12, 13). Besides, the weight for age can reflect both the gestational age and the nutritional status of children, which indicates that a well-developed body can tolerate greater impact. There have been studies showing that those who are underweight have increased risk of infection and that lower-weight premature infants are more likely to suffer from bowel disease because of malnutrition and dysbacteriosis (14, 15). Meanwhile, children with acute appendicitis suffer many serious complications if left untreated. Delayed treatment can also enhance bacterial invasion and intestinal damage (16). Interestingly enough, the regression model included total bilirubin rather than direct and indirect bilirubin. A possible explanation is that the infection not only made the erythrocyte damage increase indirect bilirubin but also influenced the enterohepatic circulation because local inflammatory stimulation causes intestinal paralysis, resulting in an increase in direct bilirubin (17, 18). Confusingly, children's age is not a high-risk factor. We assessed the correlation between months and each laboratory indicator and discovered that only the neutrophil count (*r* = 0.2, *p* = 0.028) and the neutrophil ratio (*r* = 0.551, *p* < 0.001) were statistically significant. Children's age is the main factor for the operation method (χ^2^ = 12.44, *p* = 0.014). Neutrophil count was also excluded, and the probable reason is that the inflammation indicators may fluctuate greatly in 0–3-year-olds because the number of neutrophils decreases physiologically with the increase in age, which is contrary to the increase of neutrophils during bacterial infection (19, 20).

Limited by the unobvious symptoms and cursory reword, there would be much deviation if the clinical manifestations were included (16, 21). Despite the low incidence, further multicenter trials and model validation are imperative.

In conclusion, we developed and assessed a predictive model for appendicitis that has good application value in young children (<3 years old). Pediatricians can generate the correct treatment strategy quickly based on the prediction outcomes.

## Data Availability Statement

The original contributions presented in the study are included in the article/Supplementary Material, further inquiries can be directed to the corresponding author/s.

## Ethics Statement

The studies involving human participants were reviewed and approved by Medical Ethics Committee of Shenzhen Children's Hospital. Written informed consent to participate in this study was provided by the participants' legal guardian/next of kin.

## Author Contributions

YC and ZW together completed data collection, statistical analysis, and manuscript writing. XM provided conception, design of the study, and participated in the modification of the manuscript. DX and HZ contributed to data arrangement and article revision. All authors contributed to the article and approved the submitted version.

## Conflict of Interest

The authors declare that the research was conducted in the absence of any commercial or financial relationships that could be construed as a potential conflict of interest.

## Publisher's Note

All claims expressed in this article are solely those of the authors and do not necessarily represent those of their affiliated organizations, or those of the publisher, the editors and the reviewers. Any product that may be evaluated in this article, or claim that may be made by its manufacturer, is not guaranteed or endorsed by the publisher.
